# Forensic Tools for Species Identification of Skeletal Remains: Metrics, Statistics, and OsteoID

**DOI:** 10.3390/biology11010025

**Published:** 2021-12-25

**Authors:** Heather M. Garvin, Rachel Dunn, Sabrina B. Sholts, M. Schuyler Litten, Merna Mohamed, Nathan Kuttickat, Noah Skantz

**Affiliations:** 1 Department of Anatomy, Des Moines University, Des Moines, IA 50312, USA; rachel.dunn@dmu.edu; 2 National Museum of Natural History, Smithsonian Institution, Washington, DC 20056, USA; SholtsS@si.edu (S.B.S.); littenm@si.edu (M.S.L.); 3 College of Osteopathic Medicine, Des Moines University, Des Moines, IA 50312, USA; merna.mohamed@dmu.edu (M.M.); nathan.kuttickat@dmu.edu (N.K.); noah.a.skantz@dmu.edu (N.S.)

**Keywords:** forensic anthropology, medicolegal death investigation, forensic significance, comparative osteology, human osteology, skeletal morphology, nonhuman

## Abstract

**Simple Summary:**

Forensic anthropologists are commonly asked to determine whether bones are of human origin and, if not, to which species they belong. Current practice usually relies on visual assessments rather than quantitative analyses. This study aimed to test the utility of basic bone metrics in discriminating human from nonhuman elements and assigning faunal species. A database of more than 50,000 skeletal measurements was compiled from humans and 27 nonhuman species. Equations and classification trees were developed that can differentiate human from nonhuman species with upwards of 90% accuracy, even when the bone type is not first identified. Classification trees return accuracy rates greater than 98% for the human sample. These quantitative models provide statistical support to visual assessments and can be used for preliminary assessment of a bone’s forensic significance at a scene. The statistical models, however, could not classify species at acceptable rates. For species identification, a freely available web tool (OsteoID) was created from the study data, where users can filter photographs of potential bones/species using a few basic measurements and access 3D scans and additional resources to facilitate identification. OsteoID provides an important resource for forensic anthropologists lacking access to large comparative skeletal collections, as well as other disciplines where comparative osteological training is necessary.

**Abstract:**

Although nonhuman remains constitute a significant portion of forensic anthropological casework, the potential use of bone metrics to assess the human origin and to classify species of skeletal remains has not been thoroughly investigated. This study aimed to assess the utility of quantitative methods in distinguishing human from nonhuman remains and present additional resources for species identification. Over 50,000 measurements were compiled from humans and 27 nonhuman (mostly North American) species. Decision trees developed from the long bone data can differentiate human from nonhuman remains with over 90% accuracy (>98% accuracy for the human sample), even if all long bones are pooled. Stepwise discriminant function results were slightly lower (>87.4% overall accuracy). The quantitative models can be used to support visual identifications or preliminarily assess forensic significance at scenes. For species classification, bone-specific discriminant functions returned accuracies between 77.7% and 89.1%, but classification results varied highly across species. From the study data, we developed a web tool, OsteoID, for users who can input measurements and be shown photographs of potential bones/species to aid in visual identification. OsteoID also includes supplementary images (e.g., 3D scans), creating an additional resource for forensic anthropologists and others involved in skeletal species identification and comparative osteology.

## 1. Introduction

Forensic anthropologists are commonly approached by law enforcement, coroners, and medical examiners with an unknown skeletal element and faced with a simple question: is this human [1,2] Well-trained forensic anthropologists know the human skeletal system in meticulous detail, and unless the skeletal element has been highly modified (e.g., extreme fragmentation, burning, etc.), they can usually differentiate human from nonhuman remains without hesitation [3]. Forensic anthropologists visually assess the bone, determining the element type (e.g., humerus, femur, tibia, etc.) and whether it is consistent with human anatomy based on its size (given its developmental state), shape, and bony features [3]. This macroscopic assessment is usually concluded without metric analyses.

If the bone is human, it is of forensic significance and will be subjected to a comprehensive osteological analysis. If the bone is nonhuman, a forensic anthropologist is faced with an inevitable follow-up question: what is it? This question is more than mere curiosity because it provides verifiable evidence to support the forensic anthropologist’s nonhuman designation [3]. An incorrect faunal species identification can affect the forensic anthropologist’s credibility, even if it is not of forensic importance. Similarly, responding to the inquiry by stating that it is not important or that you do not know does not instill confidence or foster positive relationships with agencies. In some cases, the animal species may provide investigators additional evidence or context regarding the circumstances of death. For example, if the remains of a cat are found intermixed with human remains, it may suggest that a suspect disposed of a house pet along with the decedent in an attempt to conceal the human remains. 

Faunal species identification, however, can be challenging for practitioners given the number of bones in a skeleton, variety of potential species, and similar morphology amongst related species [4]. While forensic anthropologists are required to be experts on the human skeleton, zooarchaeological training, while ideal, is not a requirement, and expertise in comparative osteology can vary greatly amongst practitioners. When determining the nonhuman species of skeletal remains, practitioners are fortunate if they have access to comparative osteological collections to assist with identifications. Such collections take time and resources to build or require proximity and unrestricted accessibility to an already-established collection. Various comparative osteology texts are available [5,6,7,8,9,10,11,12,13], each with their own advantages and limitations; they vary in cost, comprehensiveness, species included, photographic quality, and target audience. Texts are also most useful if the user knows the element type in advance and/or already suspects a certain species. Reliable and easily accessible online resources are limited, and internet searches for images of specific faunal elements can return mixed results.

The primary goal of this project was to develop additional, freely-available resources to support forensic anthropologists and medicolegal personnel in skeletal species identification based on simple measurements. Saulsman et al. [14] report discriminant functions derived from eight traditional long bone metrics that can differentiate human from five Australian nonhuman species with accuracy rates at or above 95%. Their sample sizes were limited to 50 human and 50 nonhuman individuals (ten per species). Given their promising results, this study aimed to test the utility of similar bone metrics in differentiating much larger samples of human and nonhuman specimens and classifying species, with a focus on species commonly encountered in North America. Although a handful of measurements cannot capture specific distinguishing bony features, traditional morphometric analyses can capture overall bone size and shape (i.e., form), which are variables considered subjectively during visual assessments of species.

In addition to the morphometric analyses, this study also aimed to develop a freely available searchable online database that uses basic metrics and visual aids (i.e., photographs and 3D scans) to help forensic anthropologists and medicolegal personnel (amongst others) determine species from skeletal elements. These resources would benefit practitioners without access to extensive comparative collections and would be accessible in the field via the use of a smart phone or other device. Beyond the scope of forensic anthropology, this skeletal species identification tool may be useful to students, archaeologists, wildlife forensic specialists, biologists, veterinarians, and others, including the general public who may wish to learn more about bones they encounter through various activities.

## 2. Materials and Methods

The study sample included skeletal data from humans and 27 faunal species frequently found in North America (20 mammals, 5 birds, 2 turtles—see Table 1), which included species that approximate human sizes (e.g., deer, horse, elk, moose, cow, pig, domestic dog, and black and brown bears). The species included are also commonly presented in comparative osteology texts used by forensic anthropologists [5,6,7,8,9] and encountered in forensic anthropological analyses [1]. To facilitate database searching, analogous measurements needed to be obtainable from each specimen included, regardless of species or element type. Thus, long bones were chosen as the main focus for this study (humerus, radius, ulna, radio-ulna, femur, tibia, fibula, and fused metapodials). For birds, the tibiotarsus was included with the tibia data, and the carpometacarpus and tarsometatarsus were included with the fused metapodials. The scapula, sacrum and os coxae were also included given the ability to take maximum lengths and breadths and their diagnostic morphologies. The original measurement list consisted of maximum lengths, proximal and distal maximum breadths (medio-lateral) and depths (antero-posterior), midshaft minimum and maximum diameters, and a few unique measurements for certain elements (e.g., femoral head diameter, acetabular diameter). Von den Driesch [15] was used as a guide when establishing the measurements.

These measurement data were collected from skeletal remains curated at the following institutions: Smithsonian National Museum of Natural History, Washington, DC; American Museum of Natural History, New York City, NY; Mercyhurst University, Erie, PA; Washburn University, Topeka, KS; University of California, Davis, CA; and Des Moines University, Des Moines, IA. Additional data were included from published papers and available datasets [16,17,18,19,20,21,22,23,24,25,26,27,28,29,30,31,32,33,34]. In some cases, published data of specimens outside of North America were included in the study to increase sample sizes if the species was the same as that commonly encountered in North America (e.g., domestic dogs and cats). Inclusion in the study required specimens to be of skeletal maturity; specimens in advanced stages of epiphyseal fusion were included to increase faunal sample sizes where necessary. This original dataset consisted of 59,442 measurements from 18,867 bones from 5207 individuals/animals). Species averages, standard deviations, and minimum/maximum ranges were calculated for each measurement. Photographs of exemplar specimens were taken from multiple standard views (e.g., six views for long bones) for incorporation into the web tool. 

A subset of the data (47,688 measurements collected from 16,315 long bone elements) was subjected to linear discriminant function (DFA) and decision tree analyses to evaluate potential methods of human versus nonhuman and species classifications (Table 1). This subset included maximum length (MaxL), maximum mediolateral width of the proximal epiphysis (MaxPW), maximum mediolateral width of the distal epiphysis (MaxDW), maximum anteroposterior depth of the distal epiphysis (MaxDD), maximum diameter of the midshaft (MaxMidD), and minimum diameter of the midshaft (MinMidD) collected from humeri, radii, ulnae, femora, and tibiae. Element-specific measurements (e.g., femoral head diameter) were excluded to permit pooled analyses across element types. Maximum proximal depth was excluded due to measurement difficulty in certain elements (e.g., tibia depending on tuberosity location, ulna, and radio-ulna). Step-wise DFA using Wilk’s lambda and a leave-one-out cross-validation were performed on the human versus pooled nonhuman samples of all long bones (replicating a situation where the element type is unknown), and then separately for each bone. DFA was used to assess human versus nonhuman classification for commonly collected univariate variables (MaxL, MaxPW, and MaxDW) and variables grouped by bone region (e.g., distal measurements and midshaft measurements) for application in cases when the unknown element is incomplete/fragmented or taphonomic modifications preclude some measurements. Finally, stepwise discriminant functions were also run to assess potential ability to classify the 28 species using both pooled-bone and bone-specific samples. Variables input into the stepwise analyses were chosen to maximize sample sizes and discriminatory power. Box’s M was used to assess homogeneity in variance–covariance matrices, and Kolmogrov–Smirnov tests were performed to evaluate data normality.

Decision trees were developed from the same data set and evaluated for classifying human versus the pooled nonhuman samples and classifying species using both the pooled-bone sample and bone-specific subsamples. The decision trees were created using a CRT (Classification and Regression Trees) growth model with a Gini impurity measure splitting criterion and a maximum tree depth of five levels. CRT uses stepwise variable selection to create a decision tree where each node is split using the variable that best maximizes the purity of the resulting nodes (i.e., homogeneity of the dependent variable) [35,36]. CRT also uses surrogate variables (those that result in a similar outcome pattern) to replace missing data, thereby maximizing sample sizes. The minimum number of cases for nodes was set at 100 for parent nodes and 50 for child nodes. Equal prior probabilities were used across groups. Tree pruning was implemented, set at one standard error in order to avoid overfitting [35,36]. A split-sample validation was applied, with the model generated from a training sample (70% of the data), which was then validated on the test sample (remaining 30% of the data). For the trees classifying human from nonhuman remains, human was set as a target variable and a misclassification cost of ten was assigned to misclassifications of human bone as nonhuman. This reflects the more severe forensic implications in erroneously assigning a human bone as nonhuman as compared to misclassifying a nonhuman bone as human.

The linear discriminant function analyses represent more traditional classification approaches but have statistical assumptions such as multivariate normality and homogeneity of variance–covariance matrices [37,38,39]. Decision trees do not rely on these statistical assumptions [40,41,42]. All statistical analyses were performed in SPSS v.28 (IBM Corporation, Armonk, NY, USA). We hypothesized that the multivariate DFA and decision trees would be able to adequately differentiate human from nonhuman remains when single elements were assessed, given that these morphometric parameters are used during visual assessments of remains. The pooled-bone sample is expected to provide less accurate results, given the compounded effects of variation within and between species and element types. The results of the DFA and decision trees were used to make informed decisions about the development of the skeletal species identification web tool, with the possibility of integrating the methods into the tool depending on their performance. 

## 3. Results

### 3.1. Descriptive Statistics

Sample sizes, minimum and maximum values, averages, standard deviations, and the ranges between two negative and two positive standard deviations (~95% confidence interval) were calculated per measurement and species (38 measurements collected across 28 species). Given the forensic aim to distinguish human from nonhuman remains, as well as the extensive dataset, Table 2 presents only the human summary statistics. This table may act as a general guide to assess whether a bone falls within the human size ranges; note, however, that there is always a small possibility of a human bone falling outside these values, given that samples may not represent the complete global variation of past and present populations. Descriptive statistics for nonhuman measurements by species are provided in the Supplementary Materials (Tables S1–S11). 

### 3.2. Morphometric Human Versus Nonhuman Classification

When the human long bone measurements are compared to those of the pooled nonhuman long bones, Box’s M indicates significant differences in the variance–covariance matrices (*p* < 0.001 for all analyses). This is true for both the pooled-bone and bone-specific samples. Kolmogrov–Smirnov results indicate that the nonhuman variables are not normally distributed, while the human data generally do not differ significantly from normality (*p* > 0.05). These results are unsurprising given the unequal sample sizes and range of nonhuman species being pooled (Table 1). DFA has been suggested to be robust against statistical violations [42]. For this reason and the exploratory nature of the analyses, the DFAs were performed despite the violation of statistical assumptions to provide comparison to the decision tree results and informed decisions about the web tool development.

The results of the human versus nonhuman DFA classification are summarized in Table 3, including overall cross-validated accuracy, group-specific cross-validated correct classifications, and sample sizes for each model. Note that DFA requires that all measurements are present for each element in the analysis, resulting in significant decreases in sample sizes for some models due to missing data. In each analysis, the cross-validated results were the same or similar to the original classification results. There are some classification biases, but in most cases, the human correct classification is higher than the nonhuman. Of the univariate analyses, maximum lengths performed the best with overall classification rates above 90% for all elements except for the ulna and a 79.5% classification rate for the pooled-bone analysis. The human classification rates using only maximum length were over 99% for all bones except the ulna (96.8%). The DFAs assessing regional measurements (two midshaft variables or two distal variables) provided results similar to or lower than the univariate maximum length results, with a few exceptions. The ulna midshaft had a 90.0% correct classification, outperforming the length results, and the humerus midshaft accuracy was much lower than the length at 67.1% (vs. 94.1% for maximum length).

As expected, the pooled-bone DFAs did not perform as well as the bone-specific analyses for morphometric human versus nonhuman classification. The pooled-bone univariate analysis of maximum distal width performed the best (87.9%), which may be because ulnae were excluded from this analysis (distal ulna measurements were not collected) thereby removing one confounding element. Maximum length correctly classified 79.5% of the sample composed of 11,129 human bones and 5254 nonhuman bones.

The multivariate stepwise DFAs returned correct human versus nonhuman classification rates above 90% for the humerus, femur, and radius and just below 90% for the tibia and ulna (Table 3). Maximum length was utilized in all the stepwise functions and had the highest weight. For the humerus (*n* = 2753) and femur (*n* = 3458), a function including maximum length and maximum distal width returned accuracy rates of 96.7% and 98.1%, respectively. Other functions for the humerus and femur returned higher classification rates (99.5% for the humerus and 99.7% for the femur), but given the variables included in these functions, sample sizes decreased to around 1100. Equations associated with the multivariate discriminate functions are provided in the Supplementary Materials (Table S12).

**Figure 1:**
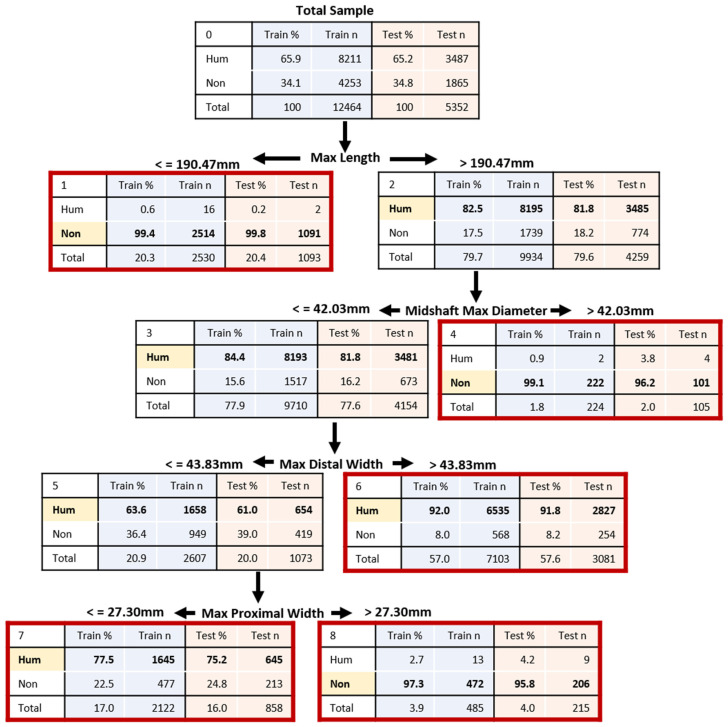
Decision tree developed to classify human (Hum) versus nonhuman (Non) elements from a pooled-bone sample (i.e., all long bones pooled). Working from the top of the tree, the variable listed at each level would be measured, and based on the provided sectioning point, the user would move down the tree to the next level. This process would continue until arriving at a terminal node where classification would be assigned. Terminal nodes are outlined in red. Group classification is highlighted in yellow and bolded at each node. Percentages and counts of bones classified to each group in the training and testing samples are presented, as well as the total percentage of the sample represented in that node. Overall correct classification for the test sample is 91.0% (99.6% for human and 75.0% for nonhuman elements). This decision tree corresponds with the first line in Table 4.

The decision tree results outperformed the DFA results for human versus nonhuman classification (Table 4) and were derived from larger samples in both the training and test sets. With all bones pooled, decision trees that evaluated all measurements correctly classified 90% or more of the training and test samples, except for the ulna test sample (89.3%). The region-specific pooled-bone analyses had lower accuracy rates (ranging from 76 to 89% correct) but still outperformed the DFA. With the exception of the ulna test sample, all training and test samples had correct human classification rates of 98% or higher. The ulna test sample correctly classified 94.5% of the human sample. Using four basic measurements, the decision tree presented in Figure 1 results in an overall accuracy of 91% and human classification accuracy of 99.6%; this is for the pooled-bone sample (i.e., without first identifying which bone is present). Although the nonhuman classification rate is lower (75%), this bias is expected given that we assigned higher misclassification costs to the human sample. The terminal nodes of the decision tree (Figure 1) indicate the number/percentage of human and nonhuman elements that fell within that node as well as associated sample sizes. Note that the “total” row depicts the percentage of the original input sample. The terminal nodes vary in their accuracy rates (75.2 to 99.8%), but only one of five terminal nodes had accuracy rates below 90%. This node (node 7) consists of ~17% of the total sample and represents those elements in which the multivariate sizes overlap between human and nonhuman species. For example, a deer metatarsal may approximate a human radius based on the measurements. Decision trees associated with the results in Table 4 are presented in the Supplementary Materials (Figures S1–S9). 

### 3.3. Morphometric Skeletal Species Identification

Correct species classification rates from the stepwise DFAs are summarized in Table 5. The pooled-bone analysis had an overall 40.4% accuracy rate, which, although better than the a priori classification rate (3.6%), can lead to numerus classification issues. For this model, 20 species had correct classification rates below 50%, with only two species (eastern cotton-tail rabbit and common box turtle) with classification rates above 75% (both above 90%). Bone-specific DFAs performed better, with overall accuracies ranging from 78 to 89%. The humerus DFA had the most accurate classifications with 18 species above 90% and none below 50%. The humerus DFA performed the worst for brown bear (55.6%), domestic dog (53.7%), and pig (50.0%). Domestic dog had classification issues across all DFAs given the high degree of variation in dog sizes and morphologies. Species within the same genus were commonly misclassified (e.g., domestic dogs and coyotes, brown bears and black bears, etc.), given their similarity in morphology and substantial overlap in body size. Human classification rates for the bone-specific DFAs ranged from 76.8% (ulna) to 100.0% (humerus, femur, and radius). All stepwise DFAs retained all variables in the final functions, and maximum length was consistently the most important variable. Ultimately, while the overall species classification rates for the bone-specific DFAs are acceptable, results varied greatly by taxa, suggesting that the DFAs should only be used as a general guide and should not be relied on as final determinants of species identification.

As might be expected, the decision tree results attempting to classify species were not successful. While tree overall classification rates were over 70% for all analyses except the ulna, none of the trees produced 28 terminal nodes to classify each species. To classify each species would require too many levels and branches; thus, the trees opted for preserving overall classification rates by focusing on those species with the highest counts. 

### 3.4. Web Tool for Species Identification

Both the DFA and decision tree results suggest that a simple equation or tree cannot be used to adequately identify skeletal species. When forensic anthropologists visually evaluate skeletal remains, they mentally process the bone dimensions to consider possible species, using the overall bone size and shape to narrow down potential species. Ultimately, however, visual comparisons and specific bony features are used to make final species identifications.

To facilitate this species identification process, we utilized the metric data and images from our study sample to develop an online, freely available species identification tool: OsteoID [43]. The home page asks users to first identify the bone, providing diverse exemplars for each element (humerus, femur, radius, radio-ulna, ulna, tibia, fibula, metapodials, scapula, sacrum, and os coxae), demonstrating the common general morphology of specific elements across most species. There is also an option to “Search All” if the user cannot confidently determine bone type. Once an option is selected, the user is brought to a new page where they can narrow the search by common name, scientific name, or by bone length, proximal width, and distal width. At any point, the user can search additional fields in the side bar. 

Maximum length, proximal width and distal width were chosen as the web tool filtering variables for several reasons. First, they were found to be the easiest to measure reliably, even with little or no osteological experience. In addition, the DFA and decision tree analyses revealed maximum length to be the most important variable in species identification, followed commonly by maximum distal width; including distal depth did not exclude many more species. Finally, the midshaft measurements are instrumentally defined (i.e., users need to take the maximum length and divide it by two to determine the correct location to take the midshaft maximum and minimum diameters) and require calipers. These factors make application in the field difficult and limit utility to those with osteological backgrounds. 

To determine the searchable range for each species/bone measurement, the minimum, maximum, and two standard deviations above and below the mean were calculated. The smallest value (whether two standard deviations below the mean or the observed minimum) was used as the lower search limit, while the largest value (either two standard deviations above the mean or the observed maximum) was used as the upper search limit. This created a conservative size range, which is important given that the dataset does not likely encompass the full size range of each species. For elements in the database missing one or more measurements, a range of 0–1000 mm was assigned so that it would not be automatically eliminated during searches. 

As possible bones/species are narrowed, thumbnails show multi-views of the bones by species as well as a list of the possible measurement ranges. Clicking on the thumbnails opens a larger image in a new window. By opening in a new window, multiple possible matches can be opened and placed side-by-side if needed. Most figures have six views of the exemplar element (anterior, posterior, medial, lateral, proximal, and distal) and include the maximum length range on the image, a scale bar, and, when possible, a penny was added for more intuitive sizing. Genus, species, collection, bone, and side information is also provided. Some images have been annotated to point out distinctive features. The user ultimately makes their final species classification based on visual comparisons. This web tool is also compatible for use on smartphones and thus is accessible in the field.

Informational tabs on the home screen describe the web tool and its development, provide instructions on utilizing the web tool (including measurement images), and answer frequently asked questions. Users are reminded that filtering the bones/species by measurements only works for skeletally mature specimens and are instructed on how to identify skeletal maturity. In numerus places, users are reminded that if a bone has any possibility of being human, they need to contact the local law enforcement agency immediately. 

Finally, a tab also refers the user to additional resources [43]. This includes references to other texts or websites as well as a link to a Dropbox folder where they can find additional project resources. In this folder, users can find the images included in the web tool, as well as images of other elements such as carpals and tarsals, which were not included in the main web tool given that measurements were not collected from these elements. Three-dimensional surface scans of many of the elements are also provided, which can be downloaded by users to view for comparison or 3D print. These 3D prints may be used to build or supplement comparative osteology collections. We are continuously expanding these Supplementary Materials and uploading them to additional digital repositories (e.g., [44]). Finally, the project data can also be accessed in this folder, as well as on Dryad [45].

## 4. Discussion

### 4.1. Human Versus Nonhuman Determination

Nonhuman remains comprise a significant portion (25–30%) of total cases assessed by forensic anthropologists [1,2,3] and can represent more than 90% of skeletal cases submitted to medical examiner offices [1]. Although forensic anthropologists mentally assess bone size and shape when determining skeletal species, only one other published study was found that assessed the utility of basic long bone osteometrics in differentiating human from nonhuman remains. Saulsman et al. [14] created discriminate functions from a sample of 50 human and 50 nonhuman specimens from five Australian species. Their study illustrated the potential utility of such quantitative methods, with accuracy rates over 95%, but it was limited by sample sizes and species inclusion. 

Our results, where more than 16,000 long bones were assessed quantitatively to develop predictive models, support their findings. From this extensive dataset, we provide discriminant functions and decision trees that can be used to assist or support human versus nonhuman determinations from long bones. Even when all elements are pooled, the DFA and decision trees return over 90% accuracy, with correct classifications of human remains over 95% (99.6% for the decision tree). Thus, high accuracy rates can be achieved even without first distinguishing the specific bony element present. If the bone is first identified and bone-specific methods are applied, accuracy increases further for all models except the tibia-specific and ulna-specific discriminant functions, which were slightly lower. The ulna performed the worst across most analyses, which may partly be due to the lack of distal measurements collected for this element. Generally, the decision tree presented slightly higher overall accuracy rates as compared to the DFAs.

When assessing the human versus nonhuman origin of skeletal remains, we recommend the use of the decision trees presented in this paper and Supplementary Materials compared to the discriminant functions, given (1) their higher accuracy rates, (2) their use of more available data and split-validation, and (3) their lack of statistical assumptions [42]. The better performance of decision trees may also reflect the incorporation of multiple sectioning points into the model (one at each node) as compared to a single sectioning point with discriminant functions. In addition, decision trees provide classification rates at each of the nodes, providing a more realistic view of accuracy and confidence in the classification for any specific set of measurements. For example, if a bone falls into the node 7 in Figure 1, the results indicate about a 75% probability that the bone is human, despite an overall model accuracy rate of 91%. Decision trees are intuitive, transparent, and easy to apply [40,41,46]. While the concept of decision trees is not new to forensic anthropology [39,40,47,48,49,50], the method remains underutilized in practice. 

Another advantage to decision tree models is that they allow users to assign higher costs to certain sets of misclassifications [36], in this case to the misclassification of human remains as nonhuman. In forensic anthropology, misclassifying human remains as nonhuman could prevent decedent identification, leaving family members without closure and impeding possible criminal investigations. In contrast, the biggest cost of misclassifying a nonhuman element as human is the unnecessary expenditure of time and resources spent in securing a scene and contacting an expert for final determination. The decision trees presented here assist in reducing the possibility of both of these scenarios. A death investigator called to a scene with a bone could have the decision tree printed on a single sheet of paper (or access it via the OsteoID website on their smartphone) and, using a tape measure, can easily follow the branches of the tree for a preliminary assessment of human versus nonhuman. Because of the integrated misclassification costs, the trees are more likely to incorrectly assign a nonhuman bone as human than vice versa; thus, the result is conservative and anything close to matching human form will be treated as if it is human and of forensic significance until determined otherwise (ideally by a trained forensic anthropologist). At the same time, resources are not wasted on scenes containing remains that are clearly not human. Thus, the models presented here can act as a triaging tool. 

While some may argue that all bones discovered should be assessed by a forensic anthropologist, this is not realistic and does not represent current practice. Forensic anthropologists typically receive elements that are believed to possibly be human. Those remains that the finder, law enforcement agent, or those consulted by the law enforcement agent (including physicians and veterinarians) deem as not human are frequently not referred to medicolegal agencies or forensic anthropologists. If referred to medicolegal agencies, their non-anthropological personnel may also determine that the remains received are not human and not worth consulting with a forensic anthropologist. Resources, such as the models and web tool presented here, can assist these individuals who are already undertaking these triaging roles to make more informed decisions. If the decision trees, discriminant functions, visual comparison with the web tool images and/or context of the remains suggest that they may be of human origin, the medicolegal agency and forensic anthropologist should be consulted for final determinations. The forensic anthropologist, in turn, may find these resources useful in supporting their designations or confirming the particular faunal species (discussed below). 

Not surprisingly, the most accurate human versus nonhuman functions and decision trees include measurements from multiple regions of the bone, which may not be possible in cases involving fragmented remains. Consequently, the use of only specific bone regions was tested as part of this study for application to larger bone fragments. Univariate analyses were performed on maximum lengths to reflect cases in which erosion to the epiphyses could affect proximal and distal elements. Models were created from only the distal measurements (width and depth) and from only the midshaft measurements (maximum and minimum diameters) for use in cases limited to these fragmented regions. The length and distal epiphyseal region-specific analyses produced higher accuracy rates than the midshaft measurements (except for the ulna). This is expected given that maximum length and distal width were commonly the most important variables in the more inclusive models. For the femoral decision tree, despite inputting all six variables, the tree output only used maximum length and was able to correctly classify over 96% of the total sample and over 99% of the human sample. The region-specific discriminate functions developed per bone (Supplemental Table S12) produced accuracy rates above 85% for all functions except the humeral midshaft (67.1%). These results are slightly higher than the region-specific DFA results presented by Saulsman et al. [14]. While the results suggest that these models may be useful tools when assessing fragmented remains as human or nonhuman, caution is still warranted given that classification rates are only moderately high, and additional evidence (e.g., presence of morphological features, application of a second method) should be provided to support the conclusion. Saulsman and colleagues [14] also warn against estimating the midshaft location on humeral fragments because deviations 2 cm above or below the actual midshaft location significantly altered their classification rates; results from femoral and tibial deviations were more robust. Application of the models to burned fragments must also consider the possibility of bone shrinkage with the thermal modification [51].

The most conservative approach for assessing the human origin of skeletal remains using osteometrics would be to compare specimen measurements with the minimum, maximum, and 95% confidence intervals for human remains presented in Table 2 and at least preliminarily consider anything that falls within that range, or very close to that range, as potentially human pending further analysis. OsteoID [43] will return images of human bones if the input measurements fall anywhere within the min/max or standard deviation ranges compiled from the sample of >2700 individuals. Practitioners must always consider the small possibility that their unknown specimen can be an outlier, perhaps lying at the extremes of the human distribution which may not have been captured in this study. Pathological conditions that affect body size (e.g., dwarfism, gigantism, etc.), although rare, could also affect results [52,53]. 

In highly fragmented or taphonomically-modified remains, morphometric and visual assessments may not be applicable. Other evidence, such as cortical bone thickness and trabecular bone density may be factored into the decision [4,54,55], although research by Rerolle et al. [56] suggests that corticomedullary index may not be as distinctive in humans as previously suggested. Several papers state that nutrient foramen location and morphology can assist in human versus nonhuman distinctions [57,58]. Microscopic (histomorphological) or molecular methods can also be utilized [59,60,61,62,63] to determine human origin, but they require greater expertise and specialized equipment, are more time intensive, and are destructive to the specimen [3]. Even histomorphological techniques cannot provide 100% accuracy in distinguishing human from nonhuman species, with certain faunal species (e.g., large mammals) and bone types (e.g., presence of only Haversian bone) shown to be particularly problematic [60]. Publications also differ on opinions of the use of osteon circularity in determining human origin of bone [62,63].

### 4.2. Species Identification

The quantitative methods of species identification were less successful than those assessing human origin. While these results are likely impacted by uneven sample sizes across the 28 species, they also reflect morphological and size similarity between some species. For example, brown bear and black bear long bones are morphologically similar [41,64,65,66,67], especially as represented by these few basic measurements; thus, small brown bears and large black bears may be misidentified. Sheep and goat long bones are also difficult to differentiate [29,68]. Domestic dogs pose many issues, not just because of their similarity to other canids included in this study (e.g., coyotes and wolves) [69,70] but also because of their high degree of variability in both morphology and size [71,72]. The DFA species classification rates were significantly higher than chance, but the probability of species misidentification remains relatively high. The application of a discriminant function to classify an unknown specimen into one of 28 groups would also be impractical to do by hand, thereby requiring computer usage. Ultimately, practitioners must rely on visual comparisons of more subtle morphological differences in making the final faunal species designations.

In facing these challenges of species identification, the OsteoID website [43] is particularly useful. Users can input basic measurements to narrow down the potential species and are presented with photographic images of the possible identifications. Thus, the measurements are used as a filtering tool, but the final identification is still based on visual comparison. With the use of visual comparisons, OsteoID can be used for identifying fragmented elements. Supplemental resources provided on the website can also be utilized in skeletal identifications, such as access to the metric database, a link to this publication and associated Supplementary Materials, 3D scans of numerous elements, and lists of other useful texts and websites. Photographs of additional elements (e.g., carpals) not included in the web tool are provided and will be continually updated. The web tool can easily be modified if future minimum/maximum values need revision. There is also the possibility of expanding the database and web tool to include additional species/specimens in the future. 

As an online, searchable, comparative osteology collection that includes photographs, data, and 3D scans, OsteoID [43] provides forensic anthropologists with a centralized location for free resources to facilitate skeletal species identification. Practitioners with less zooarchaeological training or lacking access to physical comparative collections will benefit most from these resources when determining faunal species. The web tool and online resources can be accessed from smart phones and other devices while at the scene. With the download of free third-party applications, even the 3D bone models can be viewed on smart phones. The 3D models also can be downloaded and 3D printed to create comparative collections. Beyond forensic anthropologists, forensic pathologists, medical examiners, coroners, crime scene and death investigators, and law enforcement personnel may find OsteoID useful when making preliminary assessments. In situations where scene personnel have reason to believe that remains are nonhuman and typically would have dismissed the remains as not forensically significant, they can use the OsteoID resources to visually confirm that the morphology is not consistent with a human and perhaps find a faunal species match. In cases in which there is any possibility that remains are human, expert opinions should still be obtained. Modified remains or those that are more diagnostically difficult will require a forensic anthropologist’s expertise, but OsteoID can reduce time and cost expenditures for diagnostically nonhuman remains. Bioarchaeologists, zooarchaeologists, veterinarians, and biologists may also find the OsteoID web tool and resources useful, and the general public may find interest in learning more about remains encountered. Presently, there are multiple social media groups where individuals post their skeletal finds and group participants provide species identifications. Given that OsteoID is publicly available, it contains multiple disclaimers urging anyone with remains that could potentially be human to leave them in situ and to contact local authorities. Finally, the photographs and 3D scans made available via the website can be used to train students in comparative osteology and the data may be used by researchers in other studies. 

### 4.3. Limitations and Future Directions

Given that all forensic anthropologists rely partly on bone form (i.e., size and general shape) when assessing human origin, using bone metrics to create a quantitative classification method seems simple and logical. However, our study illustrates several challenges to this work. Firstly, it is difficult to find measurements that can be collected consistently across diverse species and bones. Limiting our measurements to maximum lengths, breadths, and depths allowed us to increase the range of animals and skeletal elements in our dataset for pooled analyses, but it excludes aspects of discrete morphological features used in visual assessments of species identification. While the general morphometric variables were able to successfully differentiate human from nonhuman remains (similar to the results of Saulsman et al. [14]), visual assessments that consider specific bone features are necessary for accurate faunal species identification.

Because the methods developed here are dependent on size and epiphyseal breadths, only skeletally mature specimens could be included in quantitative analyses (and resultant functions and models are only applicable to skeletally mature specimens). At least partial fusion of both the proximal and distal epiphyses should be observed prior to utilizing the discriminant functions or decision trees. Skeletally mature specimens of certain species can be hard to locate, especially domesticated species which may be butchered as juveniles [73]. The species curated at museums vary and again tend not to focus on domesticated species or may not curate full skeletons, especially for larger mammals where space becomes a challenge. 

Unequal sample sizes from different species could have biased our classification results, particularly with human versus nonhuman analyses. Although a high degree of faunal variation is captured in the pooled nonhuman sample, there is a smaller representation of some of the largest mammalian species. Given that humans also have relatively large body sizes, this may be driving some of the classification bias, as the models may be more likely to classify all large bones (human or nonhuman) as human given the large human sample sizes. Indeed, larger animals such as moose, brown bear, horse, cow and elk were more commonly misclassified as human, which could explain the relatively higher human and lower nonhuman classification rates in the discriminant functions. Misclassifying some of these species elements as human instead of nonhuman in preliminary forensic contexts is less costly than erroneously classifying human elements as nonhuman; following the preliminary human classification, a forensic anthropologist would then be consulted for a more formal assessment that would identify the error. 

The smaller sample sizes in some nonhuman species are also less likely to capture the true population size variation and thus impact DFA species classifications. The human sample size, however, which is of greatest forensic significance, is sufficiently large, and the nonhuman sample sizes exceed those of previous publications [14]. Furthermore, not all measurements were available for all specimens. Data obtained from the literature frequently had some but not all the study measurements, meaning that in the DFAs, many of those cases were excluded. 

The species included in the metric database are not exhaustive, and it is unclear how a specimen from an excluded species would classify. This study was limited to species commonly encountered in North America that were accessible at collections but does not include, for example, marine mammals. Further validation of the developed methods is needed, and if more data can be collected from additional species and specimens, revised models may be more appropriate. Future data collection for human versus nonhuman determinations should focus on adding greater samples of larger-bodied mammals. While increased samples of larger-bodied fauna may decrease model accuracy rates, it is possible that the models may still be able to confidently differentiate human from nonhuman specimens given the distinct functional anatomy of humans [3,74,75]. 

Preliminary analyses using a subsample of the humeral and femoral data suggest that machine learning and random forest models may be able to further increase morphometric classification rates for human versus nonhuman designations and species assignments [76]. Random forest models are a machine learning approach in which numerous decision trees are created from random subsamples, and their predictions are combined through averaging to produce a final classification [46,47,48]. This machine learning technique increases classification stability and alleviates potential issues of overfitting [58]. The downside of random forest models is their complexity. Because random forest model results are based on the combined results of hundreds or thousands of trees, there is no final model/tree that can be presented or applied to cases [46]. This ensemble approach is considered a “black box” method [41] meaning that it is mathematically complex and difficult to understand and explain in terms of application [77], which can be a disadvantage in court testimony. Furthermore, for broad application, a software program would need to be created to run the random forest models with new unknown specimens.

## 5. Conclusions

The tools presented in this study do not diminish the need for forensic anthropologists. Caution must still be used given the high cost of misclassifying a human bone as nonhuman, and forensic anthropologists or other experts should be consulted in situations where there is any possibility that remains may be human. Still, the resources developed and provided here may be used to preliminarily assess whether remains are potentially human and determine the number of resources to expend on a found bone (e.g., whether or not a scene needs to be preserved, etc.). Forensic anthropologists or other medicolegal personnel can use the resources to support classifications and faunal species identifications. These resources may also be beneficial to other disciplines where skeletal remains are encountered or training in comparative osteology is beneficial, including wildlife forensics, bioarchaeology, zooarchaeology, veterinary medicine, and biology.

## Figures and Tables

**Table 1:** List of species from which data and photos were collected and sample sizes by element.

Class	Genus	Species	Common Name	Humerus	Femur	Radius	Tibia	Ulna ^1^
Aves	Anas	platyrhynchos	Mallard Duck	31	28	31	30	31
Aves	Aquila	chrysaetos	Golden Eagle	21	23	20	19	23
Aves	Branta	canadensis	Goose	34	34	32	31	34
Aves	Gallus	gallus	Chicken	31	31	31	32	31
Aves	Meleagris	gallopavo	Turkey	35	35	32	35	34
Mammalia	Alces	alces	Moose	19	17	20	21	27
Mammalia	Bos	taurus	Cow	15	16	13	17	12
Mammalia	Canis	familiaris	Domestic Dog	84	147	75	147	76
Mammalia	Canis	latrans	Coyote	64	65	57	65	58
Mammalia	Canis	lupus	Wolf	44	45	38	45	38
Mammalia	Capra	hircus	Goat	83	3	79	80	3
Mammalia	Cervus	canadensis	Elk	34	33	31	32	31
Mammalia	Didelphis	virginiana	Opossum	34	34	35	33	33
Mammalia	Ovis/capra ^2^	aries/hircus	Sheep/Goat	2	1	1	0	1
Mammalia	Equus	caballus	Horse	31	33	33	30	33
Mammalia	Felis	catus	Domestic Cat	40	39	39	39	38
Mammalia	Homo	sapiens	Human	2714	2700	2672	2684	463
Mammalia	Odocoileus	hemionus	Mule deer	31	32	34	32	38
Mammalia	Odocoileus	virginianus	White-Tailed Deer	33	39	35	39	35
Mammalia	Ovis	aries	Sheep	77	18	147	104	63
Mammalia	Procyon	lotor	Racoon	36	37	36	39	37
Mammalia	Sus	scrofa	Domestic Pig/Boar	20	17	7	17	8
Mammalia	Sylviagus	floridanus	Eastern Cotton-Tail Rabbit	36	34	34	32	33
Mammalia	Urocyon	cinereoargenteus	Gray Fox	39	42	39	40	42
Mammalia	Ursus	americanus	American Black Bear	38	34	18	19	18
Mammalia	Ursus	arctos	Brown Bear	48	46	18	22	19
Mammalia	Vulpes	vulpes	Red Fox	43	41	41	42	40
Testudines	Chelydra	serpentina	Snapping Turtle	30	30	30	30	30
Testudines	Terrapene	carolina	Common Box Turtle	31	31	27	31	31
			Totals	3778	3685	3705	3787	1360

^1^ For the human and nonhuman comparisons, individual measurements were taken from fused radio-ulna elements and included as radius or ulna. For the development of the web tool, both the individual radius and ulna measurements and combined maximum lengths/widths for the fused radio-ulna were included for search purposes. ^2^ A few specimens were labeled as “Sheep/Goat” in the collection and thus entered this way for human versus nonhuman analyses but were excluded from species analyses.

**Table 2:** Descriptive statistics from the human sample, including counts, minimums, maximums, averages, standard deviations, and two standard deviation ranges per element and measurement.

Bone	Meas ^1^	N	Min	Max	Ave	SD	−2SD	+2SD
Humerus	MaxL	2567	225	397	309	23	262	356
	MaxPD	94	35	56	46	4	37	55
	MaxPW	411	38	62	49	4	41	58
	MaxDD	94	22	37	28	3	22	35
	MaxDW	1867	42	77	59	6	47	70
	MidMaxD	425	16	32	23	3	17	28
	MidMinD	440	11	24	18	2	13	23
Radius	MaxL	2531	180	309	236	20	196	276
	MaxPD	380	15	31	23	2	18	28
	MaxPW	380	15	31	23	2	18	27
	MaxDD	89	17	36	25	4	17	32
	MaxDW	89	21	42	33	4	25	41
	MidMaxD	454	10	21	16	2	12	19
	MidMinD	454	8	74	12	3	5	19
Ulna	MaxL	406	211	334	263	20	222	303
	MaxPW	257	14	35	26	3	19	32
	MidMaxD	477	10	24	17	2	12	21
	MidMinD	477	9	19	13	2	9	17
Femur	MaxL	2630	344	550	433	33	367	499
	MaxPD	89	37	59	46	5	36	56
	MaxPW	89	71	105	87	8	72	103
	DiamH	1077	35	61	44	4	37	52
	MaxDD	89	46	92	63	7	49	78
	MaxDW	2563	58	98	77	6	64	90
	MidMaxD	457	14	39	27	3	21	33
	MidMinD	457	17	39	27	3	21	33
Tibia	MaxL	2589	159	472	357	32	294	421
	MaxPW	1867	50	94	71	6	58	84
	MaxDD	82	30	52	39	4	31	47
	MaxDW	415	40	63	52	4	43	60
	MidMaxD	420	19	44	33	4	25	42
	MidMinD	82	15	28	21	3	16	26
Fibula	MaxL	429	282	463	366	27	312	421
Os Coxae	MaxL	91	166	237	202	16	170	233
	DiamA	1526	39	63	49	4	41	57
Sacrum	MaxL	90	89	157	114	13	88	141
	MaxPW	90	90	138	111	9	93	129
Scapula	MaxL	92	127	210	178	17	145	212

^1^ Measurement abbreviations: MaxL = maximum length, MaxPW = maximum proximal width (medio-lateral), MaxDD = maximum distal depth (antero-posterior), MaxDW = maximum distal width (medio-lateral), MidMaxD = maximum diameter at midshaft, MidMinD = minimum diameter at midshaft, DiamH = femoral head diameter, DiamA = acetabulum diameter.

**Table 3:** Linear DFA accuracy results and sample sizes for human (Hum) and nonhuman (Non) classifications summarized by element and variables. Overall accuracy is bolded. Var(s) = variable(s), N_Hum_ = human sample size, N_Non_ = nonhuman sample size.

Var(s)	Pooled-Bone	Humerus	Femur	Radius	Tibia	Ulna
MaxL	**79.5%** Hum: 79.3%; Non: 80.0% N_Hum_ = 11,129; N_Non_ = 5254	**94.1%** Hum: 99.9%; Non: 78.0% N_Hu__m_ = 2567; N_Non_ = 920	**95.4%** Hum: 100.0%; Non: 83.2% N_Hum_ = 2630; N_Non_ = 981	**95.4%** Hum: 99.8%; Non: 79.3% N_Hum_ = 2531; N_Non_ = 697	**95.0%** Hum: 99.5%; Non: 83.9% N_Hum_ = 2589; N_Non_ = 1062	**78.5%** Hum: 96.8%; Non: 69.1% N_Hum_ = 406; N_Non_ = 797
MaxPW	**74.8%** Hum: 68.8%; Non: 79.2% N_Hum_ = 3383; N_Non_ = 4668	**75.4%** Hum: 85.4% Non: 69.8% N_Hum_ = 410; N_Non_ = 733	**85.4%** Hum: 100.0%; Non: 83.8% N_Hum_ = 89; N_Non_ = 822	**46.1%** Hum: 61.8%; Non: 39.7% N_Hum_ = 380; N_Non_ = 946	**93.4%** Hum: 99.7%; Non: 78.8% N_Hum_: 1867; N_Non_ = 817	**74.3%** Hum: 70.8%; Non: 76.5% N_Hum_ = 257; N_Non_ = 404
MaxDW	**87.9%** Hum: 92.9%; Non: 82.1% N_Hum_ = 5021; N_Non_ = 4345	**92.0%** Hum: 97.9%; Non: 81.2% N_Hum_ = 1868; N_Non_ = 1024	**94.8%** Hum: 100.0%; Non: 80.3% N_Hum_ = 2560; N_Non_ = 908	**69.7%** Hum: 83.1% Non: 68.1% N_Hum_ = 89; N_Non_ = 745	**89.6%** Hum: 100.0%; Non: 84.9% N_Hum_ = 415; N_Non_ = 923	--
MaxDD & MaxDW	**78.9%** Hum: 64.3%; Non: 80.0% N_Hum_ = 230; NHn = 3007	**96.3%** Hum: 100.0%; Non: 96.21% N_Hum_ = 26; N_Non_ = 850	**96.1%** Hum: 97.0%; Non: 96.1% N_Hum_ = 33; N_Non_ = 720	**86.4%** Hum: 82.0%; Non: 87.3% N_Hum_ = 89; N_Non_ = 448	**86.2%** Hum: 92.7% Non: 85.4% N_Hum_ = 82; N_Non_ = 735	--
MidMaxD & MidMinD	**64.4%** Hum: 49.0%; Non: 73.1% N_Hum_ = 1767; N_Non_ = 3089	**67.1%** Hum: 62.0%; Non: 69.9% N_Hum_ = 347; N_Non_ = 714	**90.2%** Hum: 86.4%; Non: 92.7% N_Hum_ = 457; N_Non_ = 711	**87.9%** Hum: 88.3%; Non: 87.6% N_Hum_ = 436; N_Non_ = 443	**87.2%** Hum: 84.8% Non: 87.5% N_Hum_ = 66; N_Non_ = 537	**90.0%** Hum: 94.4%; Non: 85.3% N_Hum_ = 461; N_Non_ = 428
Stepwise ^1^	**90.3%** Hum: 95.6%; Non: 87.9% N_Hum_ = 1408; N_Non_ = 3088 MaxL, MidMaxD, MidMinD	**96.7%** Hum: 99.6%; Non: 90.7% N_Hum_ = 1862; N_Non_ = 891 MaxL, MaxDW	**98.1%** Hum: 99.9%; Non: 93.0% N_Hum_ = 2552 N_Non_ = 906 MaxL, MaxDW	**91.4%** Hum: 100.0%; Non: 86.8% N_Hum_ = 327; N_Non_ = 621 MaxL, MaxPW	**89.4%** Hum: 92.2%; Non: 83.4% N_Hum_ = 1773 N_Non_ = 807 MaxL, MaxPW	**87.4%** Hum: 93.7%; Non: 77.7% N_Hum_ = 254; N_Non_ = 166 MaxL, MaxPW, MidMaxD, MidMinD

^1^ All variables were included in the stepwise DFA and those retained in the function are listed in each column with the results.

**Table 4:** Decision tree results and sample sizes for human (Hum) and nonhuman (Non) classifications summarized by element and variables. Acc = accuracy, N = sample size. See Supplementary Materials (Figures S1–S9) for the decision trees.

Bone	Input	Training Sample					Test Sample					Tree Variables
		Total Acc	Hum Acc	Non Acc	Hum N	Non N	Total Acc	Hum Acc	Non Acc	Hum N	Non N	
Pooled	All 6	91.4%	99.6%	75.4%	8211	4253	91.0%	99.6%	75.0%	3487	1865	MaxL, MidMaxD, MaxDW & MaxPW
Pooled	Distal	83.3%	98.8%	64.8%	3650	3052	82.9%	98.9%	64.5%	1495	1300	MaxDD & MaxDW
Pooled	Mid	77.2%	99.7%	61.9%	1603	2356	75.7%	99.4%	59.6%	689	1016	MidMaxD & MidMinD
Pooled	Length	88.3%	99.9%	64.5%	7696	3763	88.7%	99.8%	63.2%	3433	1491	MaxL
Humerus	ALL 6	99.1%	99.3%	98.4%	1914	741	97.9%	98.6%	96.3%	776	323	MaxL & MidMinD
Femur	ALL 6	96.7%	99.4%	89.5%	1853	694	96.4%	99.6%	86.9%	837	290	MaxL only
Radius	ALL 6	95.1%	98.6%	86.2%	1894	734	94.9%	98.6%	85.2%	778	298	MaxL & MidMaxD
Tibia	ALL 6	94.9%	98.4%	86.5%	1854	776	94.0%	97.8%	84.4%	830	327	MaxDW & MaxPW
Ulna	ALL 4	92.5%	98.7%	89.2%	318	590	89.3%	94.5%	86.5%	145	267	MidMinD & MaxPW

**Table 5:** Stepwise DFA species classification results. The right side of the table presents the number of species that fell within each accuracy range (i.e., <50%, 50–75%, 75–90%, or >90%). Vars = variables included in final function, Acc = Accuracy, Hum = human.

Bone	N	Vars	Total Acc	Hum Acc	Number of Species			
					<50%	50–75%	75–90%	>90%
Pooled	2737	All 6	40.4%	68.9%	20	6	0	2
Humerus	735	All 6	89.1%	100.0%	0	6	4	18
Femur	744	All 6	79.3%	100.0%	1	4	14	8
Radius	462	All 6	83.9%	100.0%	4	5	8	10
Tibia	548	All 6	77.7%	92.9%	3	5	5	14
Ulna	420	All 4	79.2%	76.8%	4	2	6	7

## Data Availability

The data presented in this study are openly available via the OsteoID website (www.boneidentification.com (accessed on 24 December 2021) → Additional Resources), as well as on Dryad (doi:10.5061/dryad.73n5tb2z0).

## Supplementary Materials

The following are available online at https://www.mdpi.com/article/10.3390/biology11010025/s1. Figure S1: Human versus nonhuman decision tree derived from all available measurements and a pooled-bone sample. Figure S2: Human versus nonhuman decision tree derived from only distal bone measurements and a pooled-bone sample. Figure S3: Human versus nonhuman decision tree derived from only midshaft measurements and a pooled-bone sample. Figure S4: Human versus nonhuman decision tree derived from only maximum length measurements using a pooled-bone sample. Figure S5: Human versus nonhuman decision tree for the humerus, derived from all available measurements. Figure S6: Human versus nonhuman decision tree for the femur, derived from all available measurements. Figure S7: Human versus nonhuman decision tree for the radius, derived from all available measurements. Figure S8: Human versus nonhuman decision tree for the tibia, derived from all available measurements. Figure S9: Human versus nonhuman decision tree for the ulna, derived from all available measurements. Table S1: Descriptive statistics for humeral measurements collected by species. Table S2: Descriptive statistics for femoral measurements collected by species. Table S3: Descriptive statistics for radial measurements collected by species. Table S4: Descriptive statistics for radio-ulnar measurements collected by species. Table S5: Descriptive statistics for ulnar measurements collected by species. Table S6: Descriptive statistics for tibial measurements collected by species. Table S7: Descriptive statistics for fibular measurements collected by species. Table S8: Descriptive statistics for scapular measurements collected by species. Table S9: Descriptive statistics for sacral measurements collected by species. Table S10: Descriptive statistics for pelvic measurements collected by species. Table S11: Descriptive statistics for fused metapodial measurements collected by species. Table S12: Select discriminant functions for human versus nonhuman classification.
