# Supplementary material for: Forensic Tools for Species Identification of Skeletal Remains: Metrics, Statistics, and OsteoID
Source: Biology (Basel). 2021 Dec 25;11(1):25. doi: 10.3390/biology11010025 (PMC8773354; doi:10.3390/biology11010025)
Supplement: Supplementary file 1 [file biology-11-00025-s001.zip › Supplementary Figures Garvin et al OsteoID.pdf]

Supplmentary Figures Associated with Garvin et al. (2021). Forensic approaches to species identification of skeletal re-mains: metrics, statistics, and OsteoID. *Biology*.

Supplementary Figures below represent the Decision Trees associated with Table 4 of the publication. Trees presented show training set results. Test sample classification rates and test sample size of the assigned group at each node are presented in purple text. See Table 4 for additional details regarding overall human and nonhuman accuracy rates. Note that SPSS highlights the "predicted group" at each node in grey. On a few occasions, this predicted group has lower classification rates than the other group; this is because the decision tree is making splits between the groups at each variable. The best split between nonhuman and human at some levels may still result in inacceptal classification rates. These few situations have been noted and for these specific terminal nodes we recommend indicating that the result is inconclusive. Note the following abbreviations for the decision trees: Hum = human, Non = nonhuman, MaxL = maximum length, MaxPW (MaxCPW) = maximum proximal width (medio-lateral), MaxDD = maximum distal depth (antero-posterior), MaxDW = maximum distal width (medio-lateral), MidMaxD = maximum diameter of midshaft, MidMinD = minimum diameter of midshaft. See manuscript text for additional details.

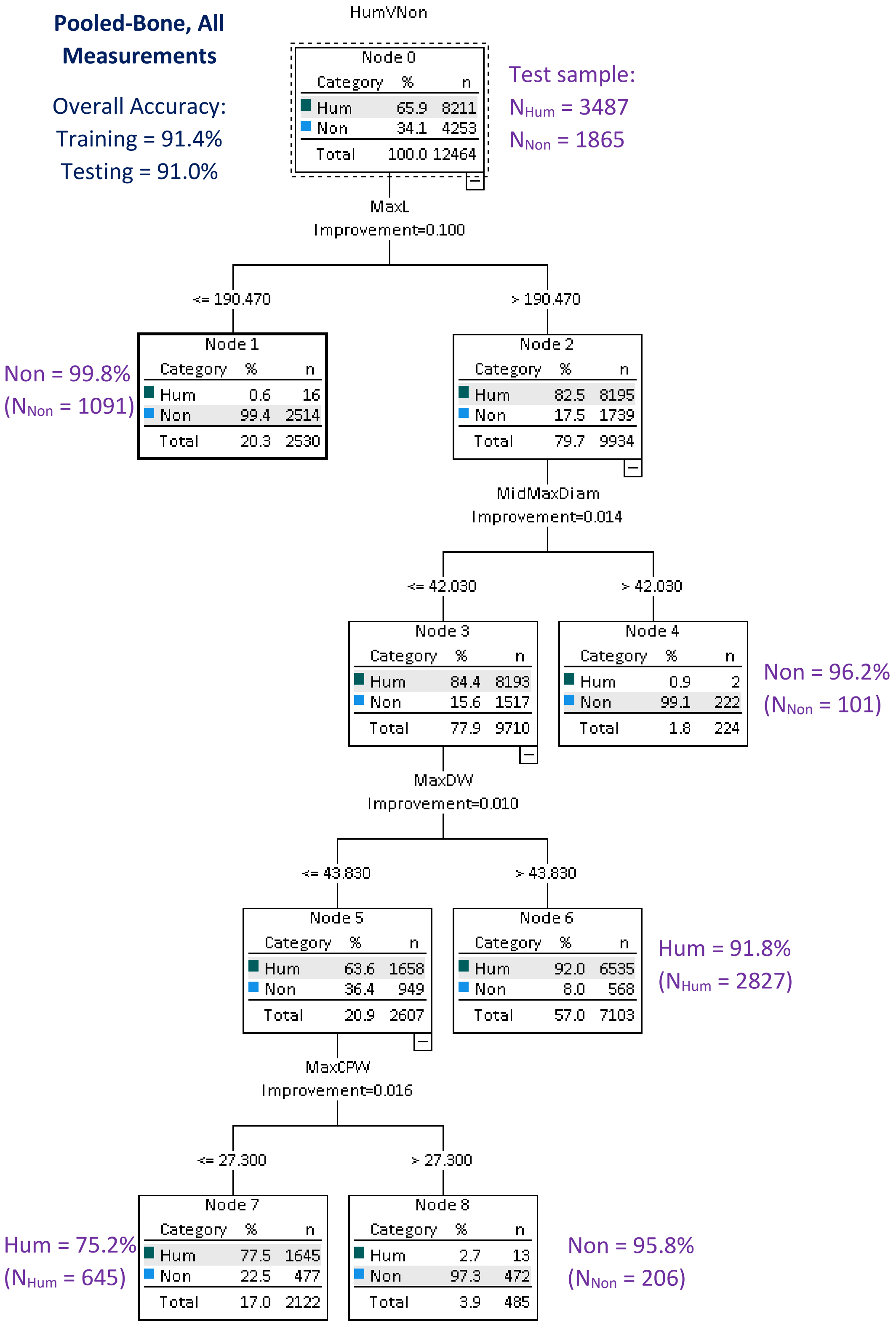

Figure S1. Human versus nonhuman decision tree derived from all available measurements and a pooled-bone sample. This tree is associated with Table 4-Line 1. Note that MaxCPW = MaxPW. This decision tree may be used on any long bone but requires length, midshaft, proximal and distal measurements. Classification percentages and counts for each group displayed at each

node. Training sample results displayed in tree. Testing sample results for the highest classifying group are noted in purple. All measurements in millimeters.

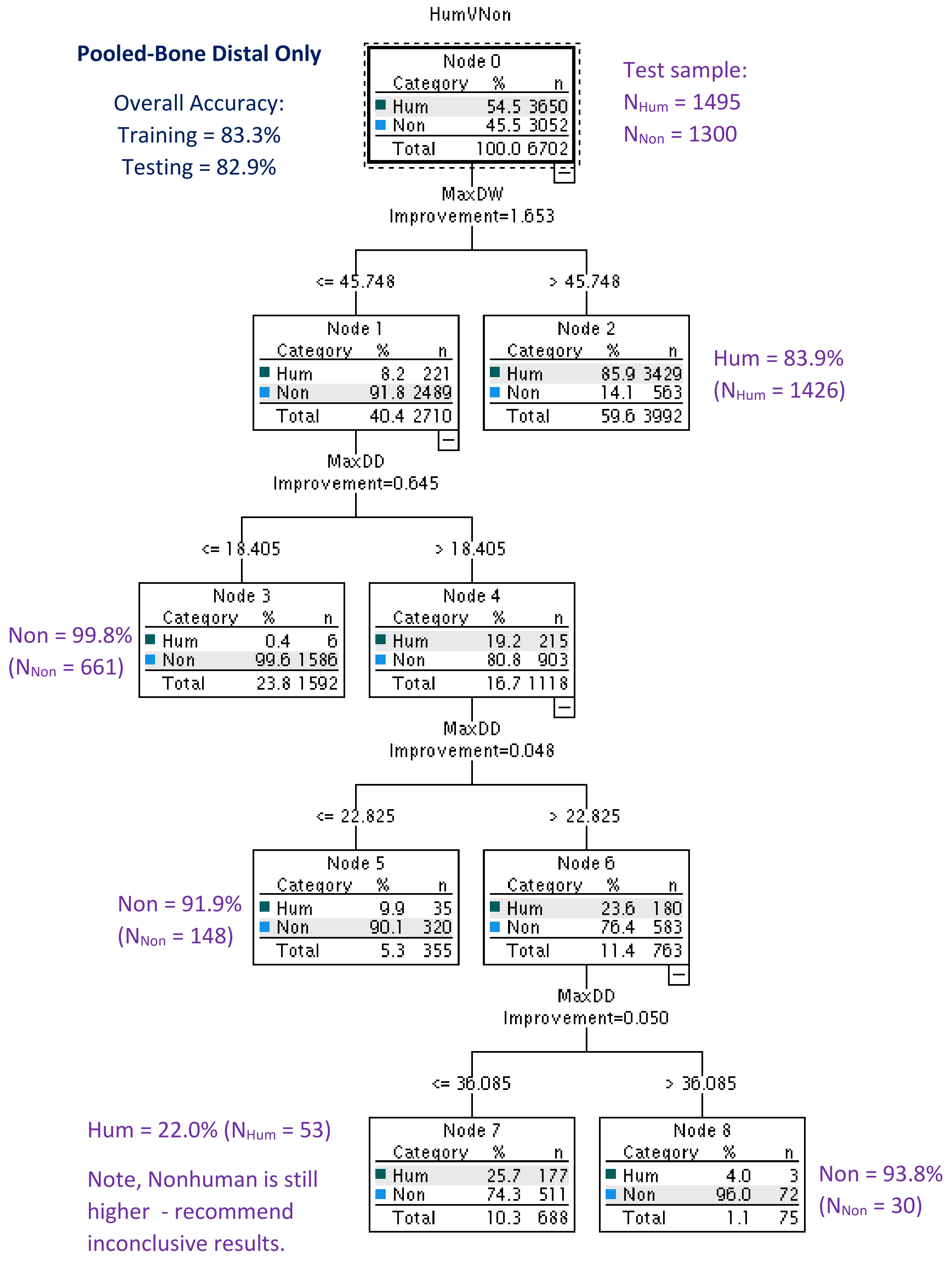

Figure S2. Human versus nonhuman decision tree derived from only distal bone measurements and a pooled-bone sample. This tree is associated with Table 4-Line 2. This decision tree may be used on a distal epiphysis of any long bone. Classification percentages and counts for each group displayed at each node. Training sample results displayed in tree. Testing sample results for the highest classifying group are noted in purple. All measurements in millimeters.

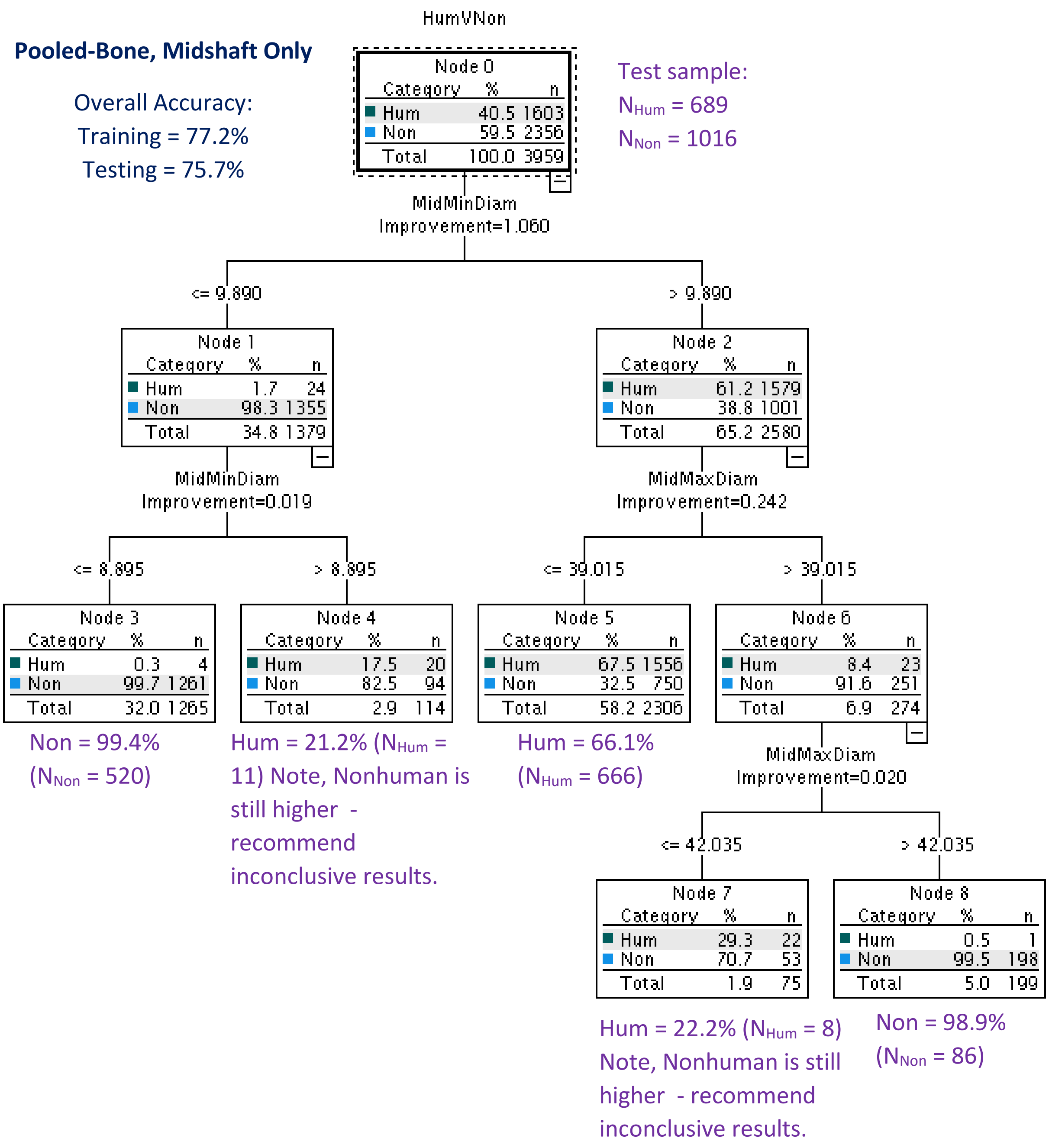

Figure S3. Human versus nonhuman decision tree derived from only midshaft measurements and a pooled-bone sample. This tree is associated with Table 4-Line 3. This decision tree may be used on a midshaft fragment of any long bone. Classification percentages and counts for each group displayed at each node. Training sample results displayed in tree. Testing sample results for the highest classifying group are noted in purple. All measurements in millimeters.

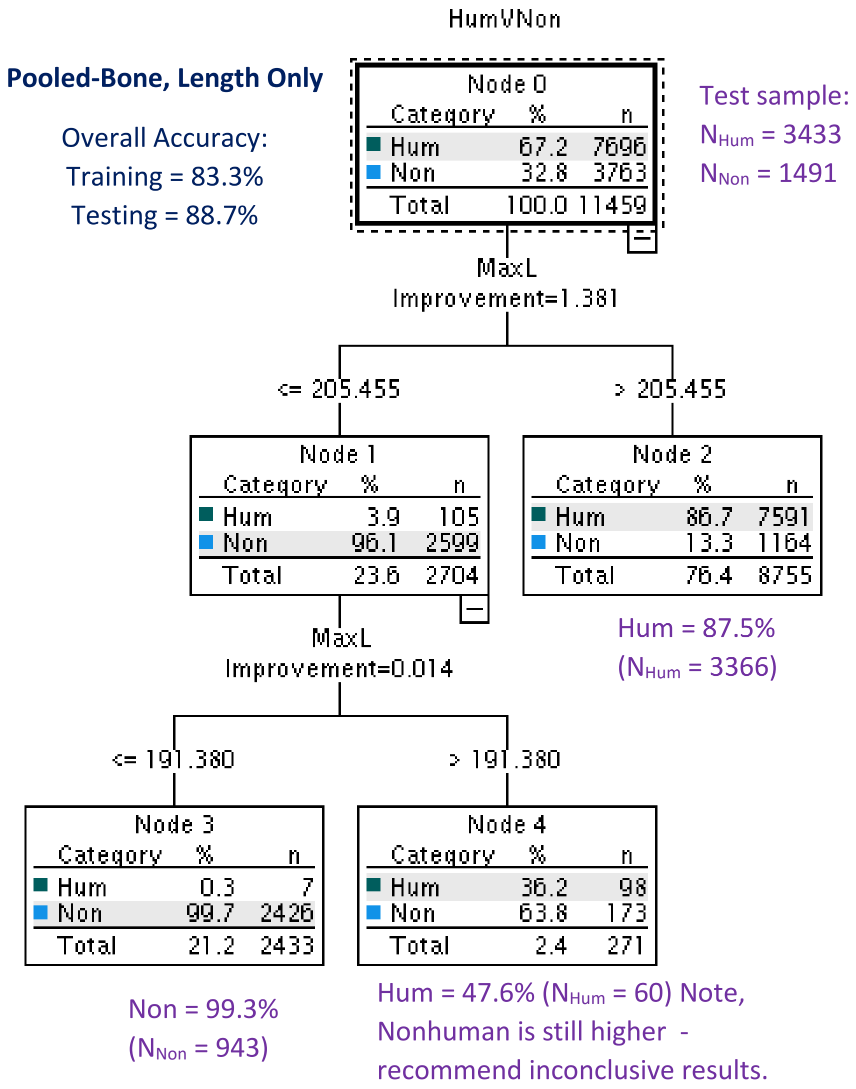

Figure S4. Human versus nonhuman decision tree derived from only maximum length measurements using a pooled-bone sample. This tree is associated with Table 4-Line 4. This decision tree may be useful if the proximal and distal epiphyses are eroded or damaged, but overall bone length is still maintained. Classification percentages and counts for each group displayed at each node. Training sample results displayed in tree. Testing sample results for the highest classifying group are noted in purple. All measurements in millimeters.

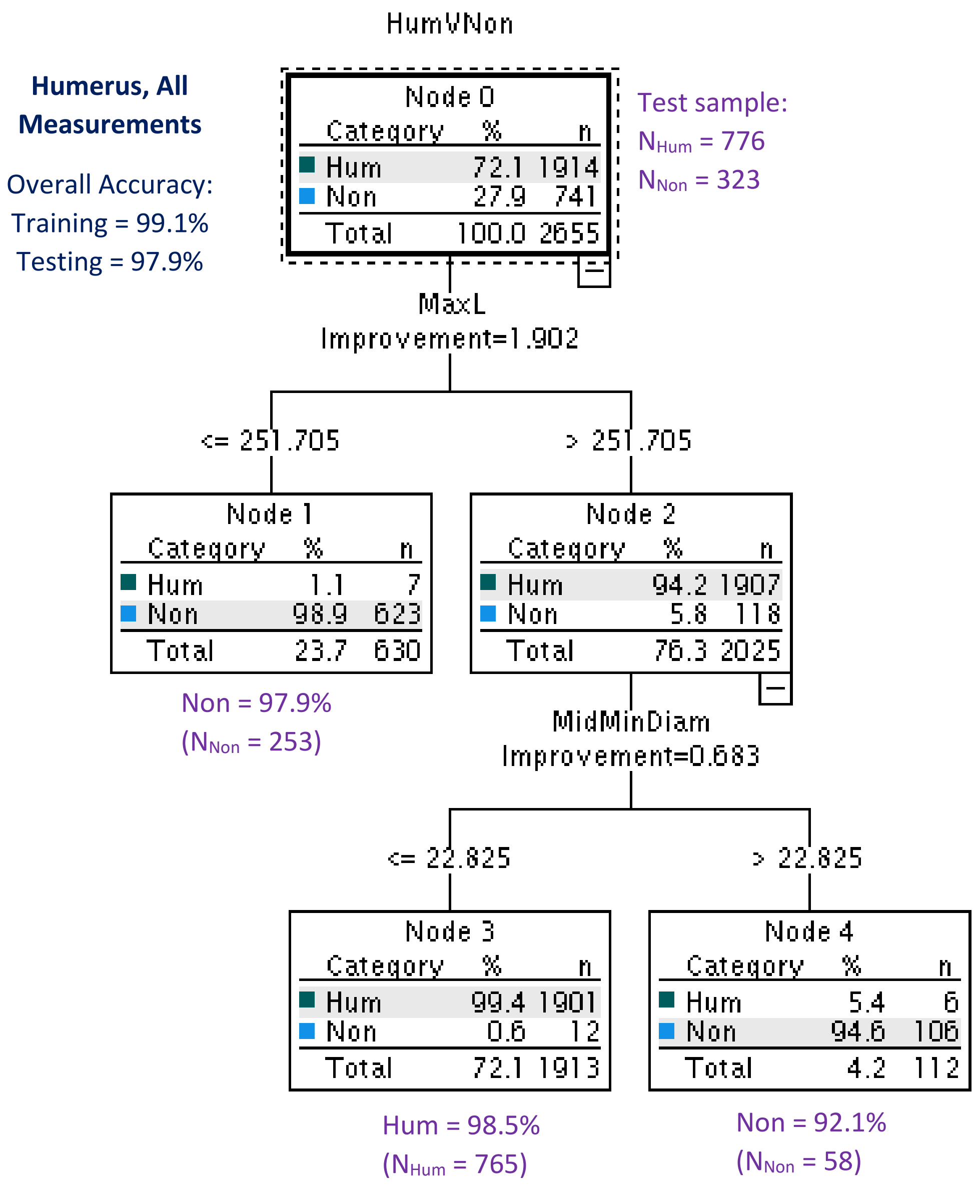

Figure S5. Human versus nonhuman decision tree for the humerus, derived from all available measurements. This tree is associated with Table 4-Line 5 and may be used if the element is able to be identified as a humerus. Classification percentages and counts for each group displayed at each node. Training sample results displayed in tree. Testing sample results for the highest classifying group are noted in purple. All measurements in millimeters.

Femur, All Measurements

Overall Accuracy:
Training = 96.7%
Testing = 96.4%

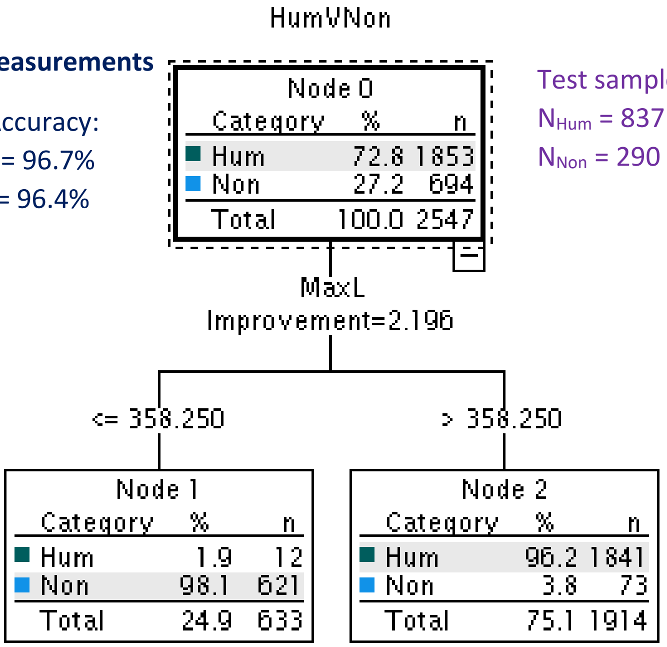

Test sample:
N_{Hum} = 837
N_{Non} = 290

Non = 98.8%
(N_{Non} = 252)

Hum = 95.6%
(N_{Hum} = 834)

Figure S6. Human versus nonhuman decision tree for the femur, derived from all available measurements. This tree is associated with Table 4-Line 6 and may be used if the element is able to be identified as a femur. Note that maximum length is the only variable required. Classification percentages and counts for each group displayed at each node. Training sample results displayed in tree. Testing sample results for the highest classifying group are noted in purple. All measurements in millimeters.

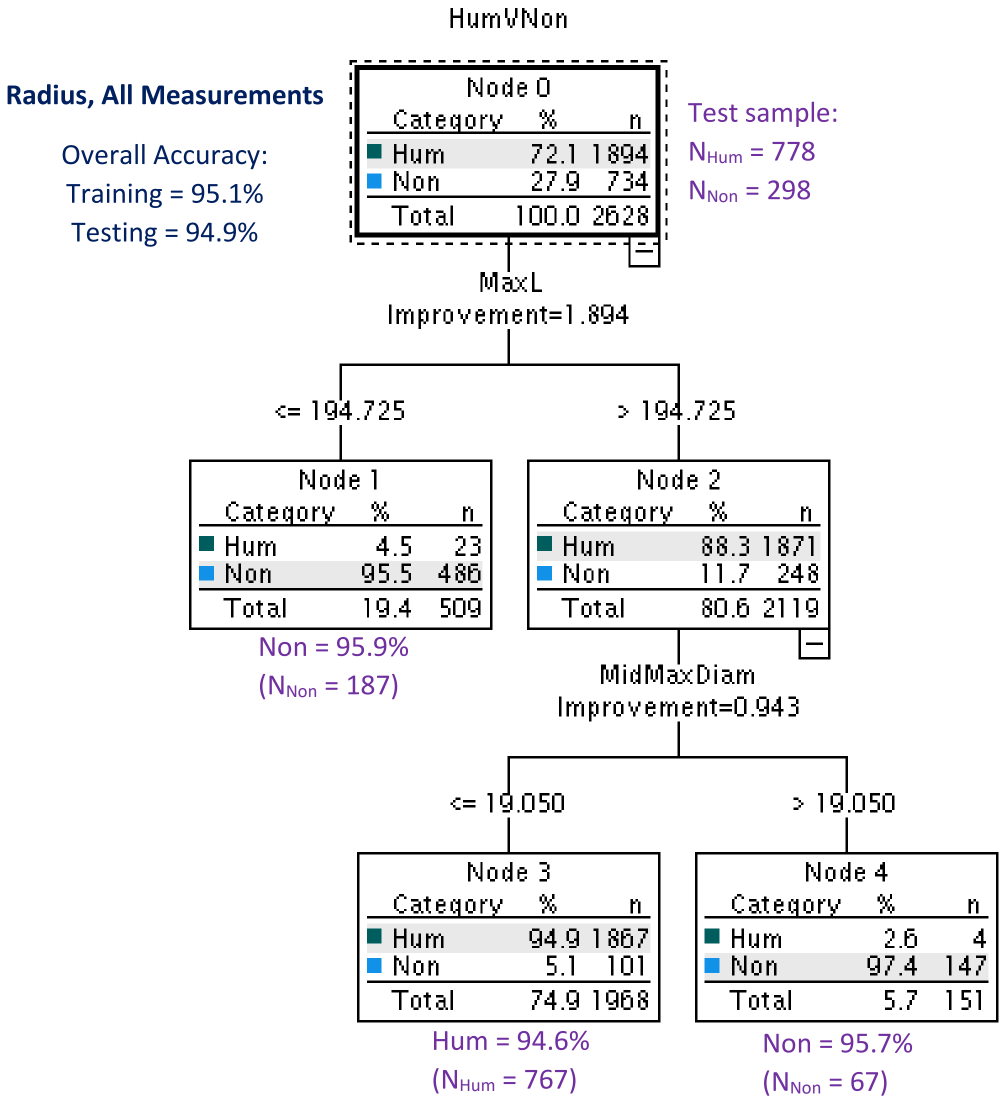

Figure S7. Human versus nonhuman decision tree for the radius, derived from all available measurements. This tree is associated with Table 4-Line 7 and may be used if the element is able to be identified as a radius. Classification percentages and counts for each group displayed at each node. Training sample results displayed in tree. Testing sample results for the highest classifying group are noted in purple. All measurements in millimeters.

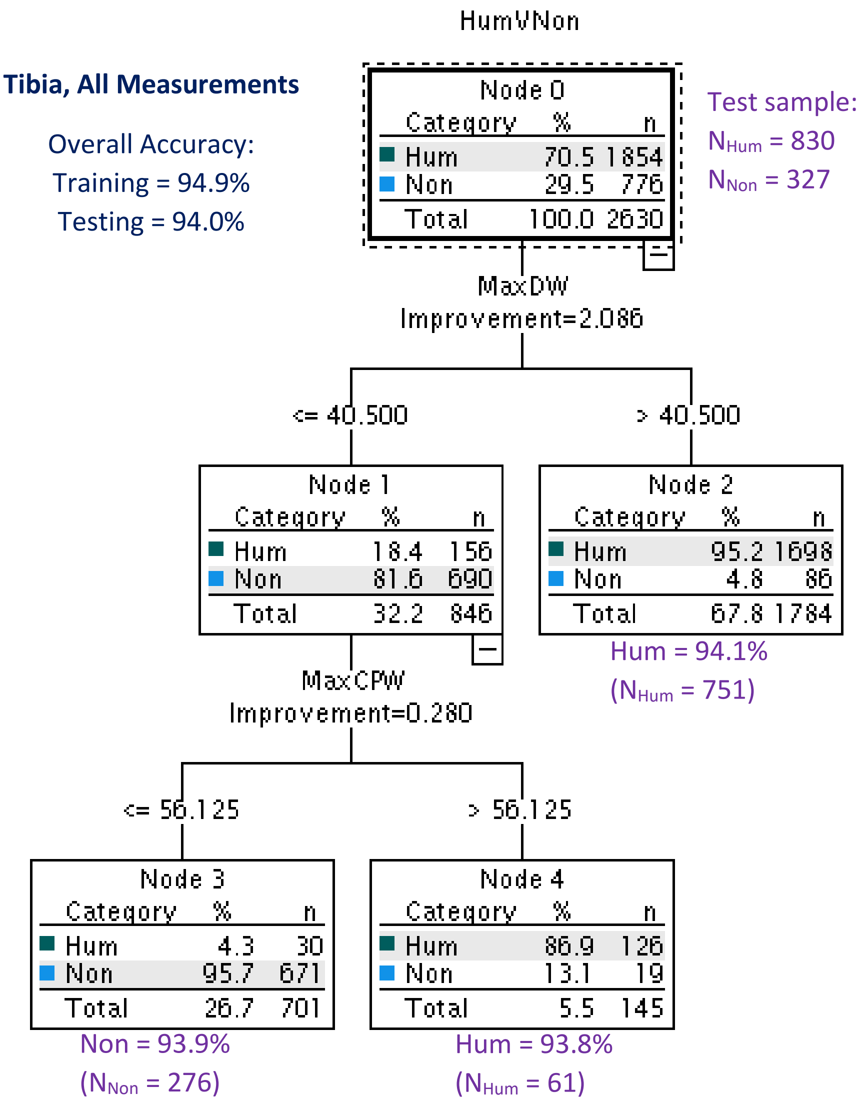

Figure S8. Human versus nonhuman decision tree for the tibia, derived from all available measurements. This tree is associated with Table 4-Line 8 and may be used if the element is able to be identified as a tibia. Note that MaxCPW = MaxPW. Classification percentages and counts for each group displayed at each node. Training sample results displayed in tree. Testing sample results for the highest classifying group are noted in purple. All measurements in millimeters.

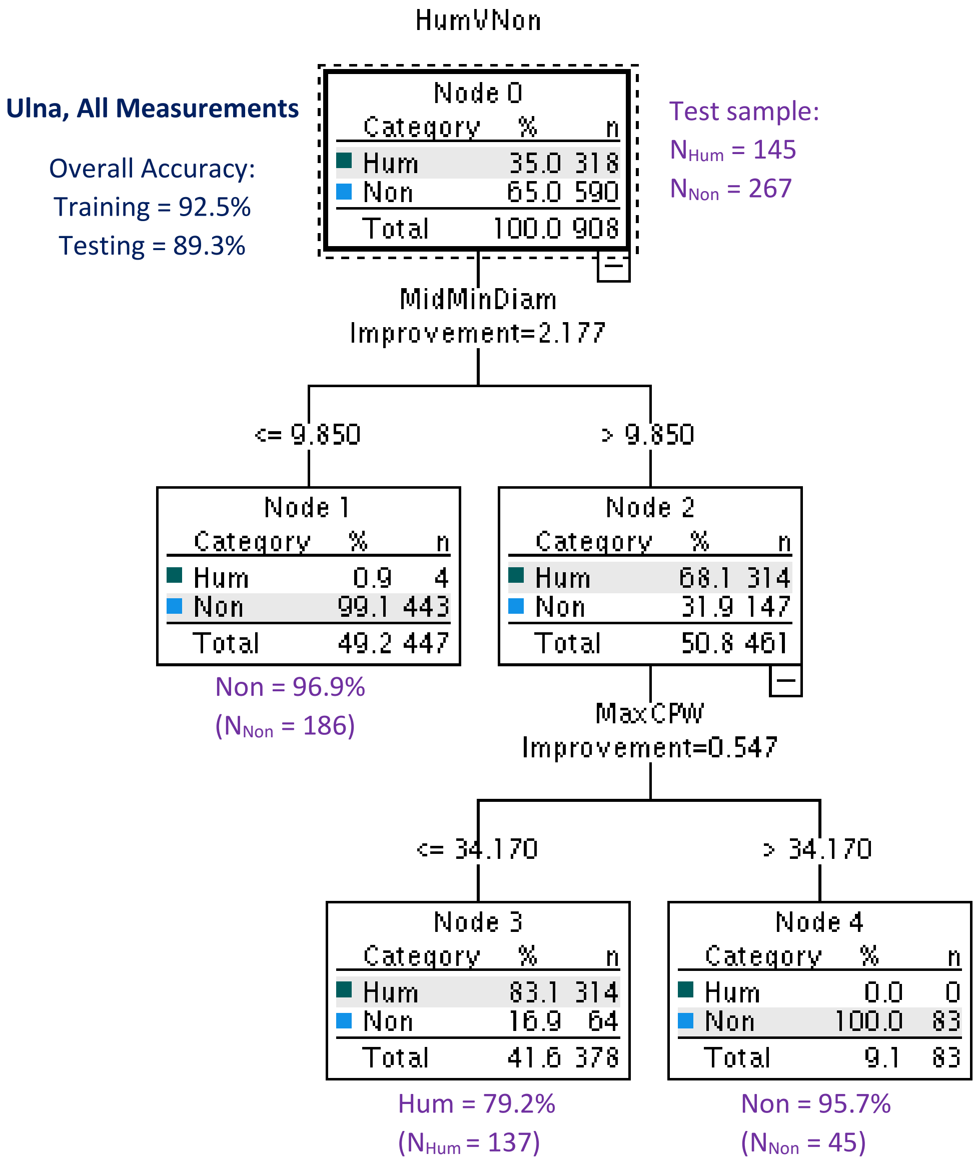

Figure S9. Human versus nonhuman decision tree for the ulna, derived from all available measurements. This tree is associated with Table 4-Line 9 and may be used if the element is able to be identified as an ulna. Note that MaxCPW = MaxPW. Classification percentages and counts for each group displayed at each node. Training sample results displayed in tree. Testing sample results for the highest classifying group are noted in purple. All measurements in millimeters.
